# Digital exclusion as a potential cause of inequalities in access to care: a survey in people with inflammatory rheumatic diseases

**DOI:** 10.1093/rap/rkac109

**Published:** 2023-01-06

**Authors:** Samantha Hider, Sara Muller, Lauren Gray, Fay Manning, Mike Brooks, Dominic Heining, Ajit Menon, Jonathan Packham, Subhra Raghuvanshi, Edward Roddy, Sarah Ryan, Ian Scott, Zoe Paskins

**Affiliations:** Haywood Academic Rheumatology Centre, Midlands Partnership Foundation Trust, Stoke-on-Trent, UK; School of Medicine, Keele University, Keele, UK; School of Medicine, Keele University, Keele, UK; Haywood Academic Rheumatology Centre, Midlands Partnership Foundation Trust, Stoke-on-Trent, UK; School of Medicine, Keele University, Keele, UK; Academic Unit of Population and Lifespan Sciences, University of Nottingham, Nottingham, UK; Haywood Academic Rheumatology Centre, Midlands Partnership Foundation Trust, Stoke-on-Trent, UK; Haywood Academic Rheumatology Centre, Midlands Partnership Foundation Trust, Stoke-on-Trent, UK; Haywood Academic Rheumatology Centre, Midlands Partnership Foundation Trust, Stoke-on-Trent, UK; Haywood Academic Rheumatology Centre, Midlands Partnership Foundation Trust, Stoke-on-Trent, UK; School of Medicine, University of Exeter, Exeter, UK; Haywood Academic Rheumatology Centre, Midlands Partnership Foundation Trust, Stoke-on-Trent, UK; Haywood Academic Rheumatology Centre, Midlands Partnership Foundation Trust, Stoke-on-Trent, UK; School of Medicine, Keele University, Keele, UK; Haywood Academic Rheumatology Centre, Midlands Partnership Foundation Trust, Stoke-on-Trent, UK; Haywood Academic Rheumatology Centre, Midlands Partnership Foundation Trust, Stoke-on-Trent, UK; School of Medicine, Keele University, Keele, UK; Haywood Academic Rheumatology Centre, Midlands Partnership Foundation Trust, Stoke-on-Trent, UK; School of Medicine, Keele University, Keele, UK

**Keywords:** digital exclusion, digital access, health literacy, digital literacy, RA, axial spondyloarthritis, PsA, inflammatory rheumatic diseases

## Abstract

**Objectives:**

COVID-19 led to rapid uptake of digital health care. We sought to examine digital access, health and digital literacy, and impact on confidence and satisfaction with remote consultations in people with inflammatory rheumatic diseases (IRDs).

**Methods:**

People with IRDs (*n* = 2024) were identified from their electronic health record and invited to participate in a cross-sectional survey, using short message service (SMS) and postal approaches. Data were collected on demographics, self-reported diagnosis, access to and use of internet-enabled devices, health and digital literacy, together with confidence and satisfaction with remote consultations. Ethical approval was obtained (Ref 21/PR/0867).

**Results:**

Six hundred and thirty-nine (639) people completed the survey [mean (s.d.) age 64.5 (13.1) years, 384 (60.1%) female]. Two hundred and eighty-seven (44.9%) completed it online. One hundred and twenty-six (19.7%) people reported not having access to an internet-enabled device. Ninety-three (14.6%) reported never accessing the internet; this proportion was highest (23%) in people with RA. One hundred and seventeen (18%) reported limited health literacy. Even in those reporting internet use, digital literacy was only moderate. People with limited health or digital literacy or without internet access were less likely to report confidence or satisfaction with remote consultations.

**Conclusion:**

Limited health and digital literacy, lack of digital access and low reported internet use were common, especially in older people with RA. People with limited health literacy or limited digital access reported lower confidence and satisfaction with remote consultations. Digital implementation roll-out needs to take account of people requiring extra support to enable them to access care digitally or risks exacerbating health inequalities.

Key messages

- Many older people with inflammatory rheumatic diseases do not have access to the internet or use it infrequently.
- Limited health and digital literacy is common and impacts on confidence and satisfaction with telemedicine.
- Clinical services need to take account of people unable to access services digitally.

## Introduction

The COVID-19 pandemic led to an overnight shift in health-care delivery and rapid uptake of digital technology. This digital transformation is supported in the National Health Service (NHS) long-term plan [1], which encourages ‘digital-first’ approaches, whereby people are encouraged to use digital tools to manage their own health, stay well and recognize important symptoms early. In parallel with the digital approach, the pandemic led to rapid adoption of remote consultations by telephone or video. Long-term adoption of some of these technologies is likely, given the perceived convenience and environmental sustainability [1, 2].

Although adoption of these technologies was by necessity at the start of the pandemic, such rapid implementation bypassed any assessment of accessibility, leading to concerns around digital access and exclusion. The Centre for Ageing Better and Citizens Online [3] explored the digital experiences of people aged 50–70 years during the pandemic using a combination of telephone and online surveys and qualitative interviews. The report recognized that there were emotional and mental health benefits from being online but concluded that the digital divide was widened during the pandemic, especially in people on low incomes who might already be at risk of poorer health outcomes.

Digital inclusion requires both digital access (to appropriate devices and reliable internet connectivity) and digital literacy (i.e. the skills, confidence and willingness to be able use the internet) to access appropriate health information. Digital or eHealth literacy describes ‘the ability to read, use computers, search for information, understand health information and put it into context’ [4]; hence, it is influenced by both general literacy levels and digital skills.

Rheumatology services need to understand more about digital inclusion among their local populations to ensure that their services are designed and delivered in ways that address the needs of all service users and do not inadvertently widen health inequalities. Given that the majority of long-term follow-up outpatient consultations (which can be informed by the use of patient-reported outcome measures, often delivered digitally) are with people who have inflammatory rheumatic diseases (IRDs), we sought to examine digital access, use, health and digital literacy, and satisfaction and confidence with remote consultations during the pandemic in people with IRDs.

## Methods

### Study design

Potential participants were identified from the rheumatology patient DIAgnostic and MONitoring Database (DIAMOND) at Midlands Partnership NHS Foundation Trust. This database contains clinical information about diagnoses, patient encounters and medications on a cohort of >20 000 patients [5]. Using the patient diagnosis term, a list of patients with a rheumatology clinician diagnosis of one of the four diagnoses of interest [RA, AS/axial spondyloarthropathy (AS/AxSpA), PsA or SLE] was assembled. Patients had to be under active follow-up (i.e. had a clinical contact within the last 2 years and had not been discharged from follow-up). Two thousand and twenty-four patients with one of the diagnoses of interest and under active follow-up were randomly selected from the database via computer. Of the 2024, providing patients had a mobile telephone number on record, they were randomly selected to be invited to participate either by via SMS text message (which included the option to complete the questionnaire via online link, email, paper or by telephone with a researcher) or postal letter, with a reminder SMS being sent at 1 week and reminder letters at 2 and 4 weeks. Those people without a mobile telephone number on their records were sent a postal invitation directly.

### Data collection

People were invited to complete a single cross-sectional questionnaire in August 2021 (to coincide with the relaxation of national COVID restrictions; for full survey, see Supplementary Data S1, available at *Rheumatology Advances in Practice* online). Data were collected on age, gender, self-reported IRD diagnosis (characterized as RA, PsA, AS/AxSpA, SLE and ‘other’), and access to and use of digital technology. People were asked to indicate whether they had access to the internet [using a checklist of basic mobile telephone, smartphone (with access to internet), computer and/or tablet] and the frequency of internet use [never, sporadically (<1 day/week), regularly (1–3 days/week), frequently (4–6 days/week) or daily]. Those reporting internet use were asked about their self-perceived ehealth literacy using the ehealth literacy scale (eHEALs) [3]. This is an eight-item measure (using a five-point Likert scale response) of eHealth literacy developed to measure people’s combined knowledge, comfort and perceived skills at finding, evaluating and applying electronic information to health problems. A higher total eHEALs indicates greater perceived digital literacy, with a score of <26 considered to represent limited digital literacy [6]. Health literacy was assessed using the single-item literacy screener [7], which asks: ‘How often do you need to have someone help you when you read instructions, pamphlets, or other written material from your doctor or pharmacy?’ Responses were dichotomized into limited (often, always, sometimes need help) and adequate (rarely, never need help) health literacy [7].

Participants were asked which services they had used to manage their arthritis during the pandemic using a checklist. They were asked to rate separately their confidence and satisfaction with talking to their rheumatology clinician on the telephone or on a video call (five options, from very satisfied/confident to very unsatisfied/confident). People were also asked about their preferences for accessing future care (telephone, video or face-to face) for a routine review appointment, for an urgent problem or for a new or first appointment about a problem. These questions were informed by our previous qualitative work, which indicated that some people had low confidence in communicating on the telephone and that preference for future consultation type was influenced by personal circumstances, such as work or family commitments [8]. Ethical approval was obtained (Surrey Borders REC Ref 21/PR/0867), and all participants provided informed consent.

Statistical analysis was performed using Stata v.17.0 (Stata Statistical Software, StataCorp LLC, TX, USA). The sample of responders was summarized using frequencies and percentages, with means and s.d. or medians and interquartile values, as appropriate. The Wilcoxon rank-sum, *t*-tests, ANOVA and chi-squared tests were used to compare continuous and categorical responses, as appropriate.

## Results

The survey was conducted in August 2021 to coincide with relaxation of England national restrictions. Six hundred and thirty-nine people completed the survey, of whom 287 (44.9%) completed it online. The mean (s.d.) age was 64.5 (13.1) years, and 384 (60.1%) were female. Six hundred and twenty-eight (98.3%) reported themselves to be of Caucasian ethnicity. The majority (492, 77%) of participants reported having RA, with 130 (20%) reporting PsA, 50 (8%) AS or AxSpA and 36 (6%) SLE or other, with 33 (5%) reporting more than one diagnosis.

One hundred and twenty-six (19.7%) responders reported no access to an internet-enabled device (Table 1), and this proportion was highest in people with RA. Those without access to an internet-enabled device were older [mean (s.d.) age 73.2 (10.5) *vs* 62.3 (12.8) years; *P* < 0.001] and less likely to be in current employment [10 (6.9%) *vs* 110 (24.1%); *P* < 0.0001].

**Table 1.** Survey demographics and internet access and use

Parameter	Total	No internet device	Internet device^c^	*P*-value
	(*n* = 612)	(*n* = 126)	(*n* = 486)	
Age group				
Age <65 years	273 (45.6)	21 (16.6)	252 (52.5)	<0.0001
Age ≥65 years	326 (54.4)	99 (78.5)	227 (46.7)	
Female gender	384 (64.7)	70 (55.5)	314 (64.6)	0.177
Employment status				
Employed	144 (24.0)	10 (7.9)	134 (27.5)	<0.0001
Other (inclucing retired)	456 (76.0)	110 (87.3)	346 (71.2)	
Arthritis diagnosis^a^				
RA	492 (77.0)	109 (86.5)	366 (75.3)	
PsA	130 (20.3)	16 (12.7)	105 (21.6)	
AS or axial SpA	50 (7.8)	8 (6.3)	39 (8.1)	
Other	23 (3.6)	4 (3.1)	19 (3.9)	
Frequency of internet use				
Never	93 (14.6)	82 (66.7)	11 (2.3)	<0.0001
<1 day/week	64 (10.5)	15 (12.2)	49 (10.1)	
1–3 days/week	68 (11.2)	8 (6.5)	60 (12.4)	
4–6 days/week	83 (13.6)	5 (4.1)	78 (16.1)	
Every day	301 (49.7)	13 (10.6)	288 (59.3)	
Limited health literacy	117 (19.3)	35 (28.7)	82 (17)	0.003
eHEALS, median (IQR)^b^	31 (25, 34)	26 (24, 32)	31 (26, 34)	0.0236
Limited digital literacy^b^	124 (26.1)	15/31 (48.4)	109/351 (24.6)	0.003
(eHEALS <26)				
Limited health and digital literacy	31 (6.6)	6 (19.4)	25 (5.7)	0.003
Sources of arthritis advice				
Websites	176 (28.8)	3 (2.3)	173 (35.6)	<0.0001
General practitioner appointment	151 (24.7)	23 (18.3)	128 (26.3)	0.061
Telephone advice line	286 (46.7)	57 (45)	229 (47.1)	0.706
Rheumatology appointment	297 (48.5)	57 (45)	240 (49.4)	0.407
Rheumatology email advice	66 (10.8)	2 (1.2)	64 (13.2)	<0.0001

Data are presented as *n* (%) unless otherwise specified.

a Arthritis diagnosis groups were not mutually exclusive because some people indicated more than one diagnosis.

b Four hundred and seventy-five patients completed eHEALS fully and could be included in the analysis. Six hundred and twelve people answered the question regarding device access.

c Internet device was defined as a smartphone or desktop or laptop computer with internet access.

eHEALs: ehealth literacy scale; IQR: interquartile range.

Three hundred and eighty-four people (63%) reported accessing the internet frequently or daily, but 93 (15.3%) reported never accessing the internet. Limited health literacy was common (*n* = 117, 19.3%). This proportion was lower in those employed [14 (10%) *vs* 101 (22.4%); *P* = 0.001] and those without internet access [35 (28.7%) *vs* 82 (16.9%); *P* = 0.003].

**Figure 1.**
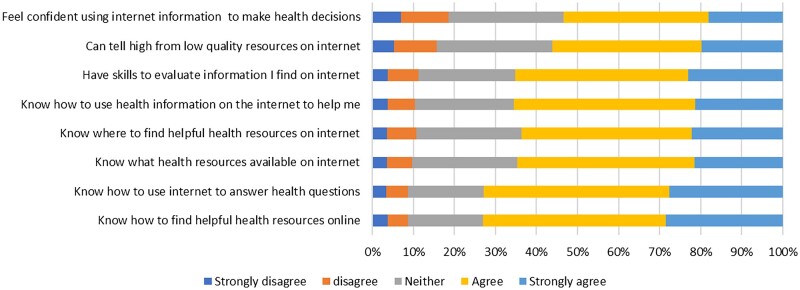
Self-reported ehealth literacy using the ehealth literacy scale

Limited digital literacy was also common, with 124 (26.1%) people reporting an eHEALS of <26, and this was reported even in people with access to an internet device. This proportion was higher in those not in employment (30.8 *vs* 13.3%; *P* < 0.0001) and ≥65 years (33.8 *vs* 19.2%; *P* < 0.0001). Furthermore, looking at the individual domains of the eHEALS (Fig. 1) illustrates that although 73% of people agreed they knew how to find health information online, slightly more than half (53%) felt confident to use information from the internet to help make health decisions.

This was reflected in the self-management strategies that people used for their arthritis, with less than one-third reporting use of websites for arthritis information (Table 1) and many using telephone helplines to manage their symptoms.

### Experiences of and preferences for remote consultations

People were asked whether their most recent rheumatology consultation was face to face (*n* = 153), telephone (*n* = 134) or video (*n* = 2). The type of consultation did not differ significantly depending on whether people had internet access or limited health or digital literacy.

Considering perceived confidence and satisfaction with remote consultations, those without internet access or who had limited health or ehealth literacy were more likely to rate themselves as being unconfident or very unconfident about both telephone and video consultations (Table 2). This was most marked for video consultations, where 41.4% of those with limited health literacy reported being unconfident or very unconfident compared with 30.3% of the survey responders. Similar findings were seen with perceived satisfaction with both telephone and video consultations (Table 2). There was no difference in confidence or satisfaction with use of telephone consultations in relationship to age or employment status. Older age and not being employed were associated with less perceived confidence and satisfaction with video consultations. People with limited health literacy or limited digital literacy were less confident and less satisfied with both types of remote consultations than people with adequate health and digital literacy. Gender was not associated with confidence or satisfaction with either telephone or video calls.

**Table 2.** Impact of internet access, health and ehealth literacy on confidence and satisfaction with remote consultations

Parameter	Internet access (*n* = 612)		Limited health literacy (*n* = 606)		Limited digital literacy (*n* = 475)		Total
	No	Yes	Yes	No	Yes	No	
	(*n* = 126)	(*n* = 486)	(*n* = 117)	(*n* = 489)	(*n* = 124)	(*n* = 351)	
Most recent rheumatology consultation							
Telephone	24 (46.2)	110 (46.6)	24 (49.0)	109 (46.4)	22 (41.5)	82 (46.6)	134 (46.5)
Video	0	1 (0.4)	0	1 (0.4)	0	1 (0.6)	1 (0.4)
Face to face	28 (53.9)	125 (53.0)	25 (51.0)	125 (53.2)	31 (58.5)	93 (52.8)	153 (53.1)
Confidence for a telephone consultation							
Very confident	33 (27.7)	178 (37.1)	27 (23.3)	181 (37.9)	39 (31.7)	132 (37.9)	211 (35.2)
Confident	51 (42.9)	194 (40.4)	41 (35.3)	20 4(42.7)	42 (34.2)	153 (44.0)	245 (40.9)
Neither	11 (9.2)	48 (10.0)	16 (13.8)	42 (8.8)	19 (15.5)	28 (8.1)	59 (10.0)
Unconfident	18 (15.1)	50 (10.4)	27 (23.3)	40 (8.4)	19 (15.5)	29 (8.3)	68 (11.4)
Very unconfident	6 (5.0)	10 (2.1)	5 (4.3)	11 (2.3)	4 (3.3)	6 (1.7)	16 (6.7)
Confidence for a video consultation							
Very confident	7 (6.9)	106 (22.5)	15 (13.5)	98 (21.3)	8 (6.6)	92 (26.7)	113 (19.7)
Confident	22 (21.6)	166 (35.2)	32 (28.8)	155 (33.7)	36 (29.8)	133 (38.7)	188 (32.8)
Neither	22 (21.6)	77 (16.3)	18 (16.2)	81 (17.6)	32 (26.5)	45 (13.1)	99 (17.3)
Unconfident	30 (29.4)	89 (18.9)	31 (27.9)	87 (18.9)	32 (26.5)	53 (15.4)	119 (20.7)
Very unconfident	21 (20.6)	34 (7.2)	15 (13.5)	39 (8.5)	13 (10.7)	21 (6.1)	55 (9.6)
Satisfaction for a telephone appointment							
Very satisfied	32 (27.6)	139 (29.1)	23 (20.0)	145 (30.6)	24 (19.7)	107 (30.8)	171 (28.8)
Satisfied	41 (35.3)	198 (41.4)	41 (35.7)	198 (41.8)	38 (31.2)	149 (42.9)	239 (40.2)
Neither	16 (13.8)	59 (12.3)	19 (16.5)	56 (11.8)	27 (22.1)	37 (10.7)	75 (12.6)
Unsatisfied	20 (17.2)	68 (14.2)	22 (19.1)	64 (13.5)	25 (20.5)	46 (13.3)	88 (14.8)
Very unsatisfied	7 (6.0)	14 (2.9)	10 (8.7)	11 (2.3)	8 (6.6)	8 (2.3)	21 (3.5)
Satisfaction for a video appointment							
Very satisfied	6 (6.0)	79 (16.7)	11 (9.9)	74 (16.1)	8 (6.6)	69 (20.1)	85 (14.8)
Satisfied	18 (18.0)	168 (35.5)	25 (32.5)	150 (32.7)	34 (27.9)	127 (37.0)	186 (32.5)
Neither	30 (30.0)	108 (22.8)	28 (25.2)	110 (24.0)	35 (28.7)	77 (22.5)	138 (24.1)
Unsatisfied	22 (22.0)	83 (17.6)	21 (18.9)	83 (18.1)	31 (25.4)	49 (14.3)	105 (18.3)
Very unsatisfied	24 (24.0)	35 (7.4)	16 (14.4)	42 (9.2)	14 (11.5)	21 (6.1)	59 (10.3)

The majority of people preferred face-to-face consultations in the future, although for urgent problems (such as an arthritis flare) or for a routine review appointment more people would consider a telephone consultation than would consider this for a new/first appointment about a problem (Table 3). Those preferring a telephone consultation were similar to those preferring video, face-to-face or a choice at the time in terms of age, gender, employment status and health literacy. The same pattern was seen across IRD groups.

**Table 3.** Impact of internet access, health and ehealth literacy on preferences for future care

Parameter	Internet access		Limited health literacy		Limited digital literacy		
	No	Yes	Yes	No	Yes	No	Total
	(*n* = 126)	(*n* = 486)	(*n* = 117)	(*n* = 489)	(*n* = 124)	(*n* = 351)	
Preference for future first appointment/new problem							
Telephone	19 (15.8)	46 (9.5)	12 (10.3)	52 (10.9)	7 (5.7)	40 (11.5)	65 (10.8)
Video	0	7 (1.5)	1 (0.9)	6 (1.3)	0	7 (2.0)	7 (1.2)
Face to face	81 (67.5)	328 (68.1)	87 (74.4)	318 (66.4)	88 (72.1)	231 (66.2)	409 (67.9)
Choice at the time	20 (16.7)	101 (21.0)	17 (14.5)	103 (21.5)	27 (22.1)	71 (20.3)	121 (20.1)
Preference for future urgent problem							
Telephone	25 (21.4)	80 (16.5)	20 (17.1)	84 (17.5)	14 (11.4)	64 (18.3)	105 (17.5)
Video	0	11 (2.3)	2 (1.7)	9 (1.9)	1 (0.8)	10 (2.9)	11 (1.8)
Face to face	77 (65.8)	294 (60.7)	78 (66.7)	290 (60.5)	85 (69.1)	201 (57.4)	371 (61.7)
Choice at the time	15 (12.8)	99 (20.5)	17 (14.5)	96 (20.0)	23 (18.7)	75 (21.4)	114 (19.0)
Preference for future routine review appointment							
Telephone	35 (29.4)	125 (25.9)	25 (21.4)	134 (28.0)	22 (17.9)	95 (27.2)	160 (26.6)
Video	1 (0.8)	17 (3.5)	2 (1.7)	16 (3.3)	1 (0.8)	17 (4.9)	18 (3.0)
Face to face	67 (56.3)	236 (48.9)	74 (63.3)	224 (46.8)	72 (58.5)	161 (46.1)	303 (50.3)
Choice at the time	16 (13.5)	105 (21.7)	16 (13.7)	105 (21.9)	28 (22.8)	76 (21.8)	121 (20.1)

## Discussion

This survey demonstrates that in a population of people with IRDs, one in five people do not have access to an internet-enabled device and 15% report never using the internet. Furthermore, even in patients who have access to an internet-enabled device up to one in five people report limited health or digital literacy. People without access to the internet or with limited health or digital literacy are also less likely to be confident or satisfied with remote consultations. This might be a significant barrier to longer-term uptake of remote consultations. Given that the NHS Long-Term plan [1] is moving to ‘digital by default’, this risks digital exclusion and widening inequalities for people without access to or confidence with digital technology [9–11]. This is a particular concern for people with IRDs, who are high users of health and social care [12].

Although we studied patients attending a single secondary care centre, potentially limiting generalizability (especially regarding ethnicity), our results are similar to general population data suggesting that the UK digital divide is influenced by age, socioeconomic status and whether a person has a disability [13]. Stoke-on-Trent has high levels of socioeconomic deprivation [13] and significant levels of limited health literacy [14], both of which might impact negatively on digital access and skills. However, it is possible that our figures might underestimate the problem, because people with limited literacy might be less likely to complete surveys, although we attempted to mitigate this by offering multiple methods of survey completion, including by telephone with a researcher. We identified that one in five responders had limited health literacy, which is similar to other studies [7]. However, although the single-item screener for health literacy performs moderately well at identifying those with limited reading difficulty [7], it might identify less than half of people who lack competency to interpret and understand written health information [15].

In common with general population data [13], we found that with increasing age, internet access and usage decreases: 100% of respondents in the 16–34-year age group reported going online daily or almost daily, compared with only 67% in the 65+-year age group. However, surveys of internet use in older people suggest that although internet use increased significantly during the pandemic, many older people or those on low incomes remained offline, and of these, many felt that they did not need to be able to use the internet and valued non-digital approaches [3]. Thus, although the sociodemographics of our area might mean that the rates of digital exclusion are higher than in other areas, the impact of age on digital exclusion still needs to be considered when developing services to prevent worsening health inequalities in people at highest risk of poor outcomes [12].

Most previous studies in this area have focused on people with RA. A French multicentre, cross-sectional study [16] showed that 82% of participants had digital access (compared with 77% of our cohort with RA), although only 29% reported using it specifically for RA-related reasons. In contrast, a single-centre German study of people with inflammatory arthritis showed that although 91% of their cohort had access to a smartphone and 75% reported accessing the internet for health information, eHealth literacy was low [17]. Our survey is novel in including a broader range of IRDs than RA.

Our digital literacy results are similar to those seen in an international cross-sectional study of people with poorly controlled RA [18], with limited digital literacy being seen in nearly two-thirds of people and only one-third reporting that they found the internet useful to help inform decisions about their health. In contrast, a Canadian study of adults aged >50 years with a recent fracture [19] showed that although digital access was similar, levels of digital literacy were higher, and a significant proportion reported using the internet to look for medical information for themselves or others. Thus, although the single-centre nature of our results is a weakness and limits generalizability, comparison of our results with other published cohorts provides confidence in our findings. A strength of our findings is the broad recruitment strategy, enabling people to participate either online or via a paper survey (with 56% completed as hard copy) in addition to the use of validated tools (such as eHEALs [4]) for examining digital literacy.

Reflecting other published data on telemedicine in rheumatology [20], our results suggest a strong preference for face-to-face appointments, especially for first appointments [20], although our responders were more likely to consider telephone appointments for routine reviews or urgent appointments. This study adds to the findings of Sloan *et al.* [20] in two ways. First, our respondents expressed a preference for telephone over video consultations, which might be reflective of the level of health literacy in our population. Second, one in five people reported wanting to have a choice of consultation modality at the time of the appointment. The notion that patient preferences for remote consultations vary depending on their context and situation at the time of appointments was highlighted in our previous qualitative studies [8] and has important ramifications for service design.

To our knowledge, this is the first UK study to examine the impacts of internet access and of digital and health literacy with preferences and confidence for telemedicine. Although digital access and literacy skills did not seem to influence preferences for a particular consultation type, our results suggest that people without digital access or with limited health literacy were likely to report perceived lower confidence and satisfaction with both telephone and video consultations. In a small US study, health literacy was not associated with willingness to undertake video consultations, although their population was younger than our cohort and all reported access to an internet-enabled device [21]. Nearly 2 million people in the UK alone report being unable to explain symptoms and feelings on the telephone, and our qualitative studies also identified that in addition to reduced confidence in being able to talk on the telephone, some participants described conversations as stressful and more hurried [8, 22]. Given that people without access to the internet or with limited health literacy are more likely to be socioeconomically deprived and therefore at risk of poorer health outcomes [23], it is important to increase awareness and address these factors in health-care delivery to prevent worsening of existing health inequalities.

In summary, our results emphasize the challenge of digital inclusion for people with IRDs and demonstrate that even with digital access, people might need support to enhance their digital literacy and skills to support more effective telemedicine consultations and promote digital arthritis self-management. Service providers need to consider the impact of digital exclusion and support efforts to enable people to access care digitally where appropriate, whilst considering patient preferences and continuing to provide alternative non-digital ways for people to access care services for those not online.

## Supplementary Material

rkac109_Supplementary_Data

## Data Availability

The data underlying this article will be shared on reasonable request to the corresponding author.

## References

[1] NHS. NHS long term plan. https://www.longtermplan.nhs.uk/online-version/chapter-5-digitally-enabled-care-will-go-mainstream-across-the-nhs/ (4 January 2023, date last accessed).

[2] Royal College of Physicians. Outpatients: the future – adding value through sustainability. London: RCP, 2018.

[3] Centre for Ageing Better/Citizens Online. Digital skills to connect report. 2021. https://ageing-better.org.uk/sites/default/files/2021-07/Digital-Skills-to-Connect.pdf (4 January 2023, date last accessed).

[4] Norman CD , Skinner HA. eHEALS: the eHealth literacy scale. J Med Internet Res 2006;8:e27.doi: 10.2196/jmir.8.4.e27. 17213046 PMC1794004

[5] Grove ML , Hassell AB, Hay EM, Shadforth MF. Adverse reactions to disease-modifying anti-rheumatic drugs in clinical practice. QJM 2001;94:309–19. doi: 10.1093/qjmed/94.6.309. 11391029

[6] Richtering SS , Hyun K, Neubeck L et al eHealth literacy: predictors in a population with moderate-to-high cardiovascular risk. JMIR Hum Factors 2017;4:e4.doi: 10.2196/humanfactors.6217. 28130203 PMC5303199

[7] Morris NS , MacLean CD, Chew LD et al The Single Item Literacy Screener: evaluation of a brief instrument to identify limited reading ability. BMC Fam Pract 2006;7:21.doi: 10.1186/1471-2296-7-21. 16563164 PMC1435902

[8] Paskins Z , Bullock L, Manning F et al Acceptability of, and preferences for, remote consulting during COVID-19 among older patients with two common long-term musculoskeletal conditions: findings from three qualitative studies and recommendations for practice. BMC Musculoskelet Disord 2022;23:312.doi: 10.1186/s12891-022-05273-1. 35366845 PMC8976169

[9] Fang ML , Canham SL, Battersby L et al Exploring privilege in the digital divide: implications for theory, policy, and practice. Gerontologist 2019;59:e1–15. doi: 10.1093/geront/gny037. 29750241

[10] Turner A , Morris R, Rakhra D et al Unintended consequences of online consultations: a qualitative study in UK primary care. Br J Gen Pract 2022;72: e128–37. doi: 10.3399/BJGP.2021.0426. 34903520 PMC8813120

[11] Sheikh A , Anderson M, Albala S et al Health information technology and digital innovation for national learning health and care systems. Lancet Digital Health 2021;3:e383–96. 33967002 10.1016/S2589-7500(21)00005-4

[12] Versus Arthritis. The State of Musculoskeletal Health 2021. https://www.versusarthritis.org/media/24238/state-of-msk-health-2021.pdf (4 January 2023, date last accessed).

[13] Office for Nataional Statistics. Office for National Statistics Survey 2020. https://www.ons.gov.uk/peoplepopulationandcommunity/householdcharacteristics/homeinternetandsocialmediausage/bulletins/internetaccesshouseholdsandindividuals/2020 (4 January 2023, date last accessed).

[14] Protheroe J , Whittle R, Bartlam B et al Health literacy, associated lifestyle and demographic factors in adult population of an English city: a cross-sectional survey. Health Expect 2017;20:112–9. doi: 10.1111/hex.12440. 26774107 PMC5217902

[15] Rowlands G , Protheroe J, Winkley J et al A mismatch between population health literacy and the complexity of health information: an observational study. Br J Gen Pract 2015;65:e379–86. 26009533 10.3399/bjgp15X685285 PMC4439828

[16] Magnol M , Eleonore B, Claire R et al Use of eHealth by patients with rheumatoid arthritis: observational, cross-sectional, multicenter study. J Med Internet Res 2021;23:e19998. 33512320 10.2196/19998 PMC7880811

[17] Knitza J , Simon D, Lambrecht A et al Mobile health usage, preferences, barriers, and eHealth literacy in rheumatology: patient survey study. JMIR mHealth and uHealth 2020;8:e19661. 32678796 10.2196/19661 PMC7450373

[18] Taylor PC , Ancuta C, Nagy O et al Treatment satisfaction, patient preferences, and the impact of suboptimal disease control in a large international rheumatoid arthritis cohort: SENSE study. Patient Prefer Adherence 2021;15:359–73. doi: 10.2147/PPA.S289692. 33633444 PMC7900444

[19] Cherid C , Baghdadli A, Wall M et al Current level of technology use, health and eHealth literacy in older Canadians with a recent fracture—a survey in orthopedic clinics. Osteoporos Int 2020;31:1333–40. doi: 10.1007/s00198-020-05359-3. 32112118

[20] Sloan M , Lever E, Harwood R et al Telemedicine in rheumatology: a mixed methods study exploring acceptability, preferences and experiences among patients and clinicians. Rheumatology (Oxford) 2022;61:2262–74. doi: 10.1093/rheumatology/keab796. 34698822 PMC8689882

[21] Dekker AB , Bandell DLJI, Kortlever JTP, Schipper IB, Ring D. Factors associated with patient willingness to conduct a remote video musculoskeletal consultation. Arch Bone Jt Surg 2020;8:656–60. 33313344 PMC7718570

[22] Patient Information Forum. Health literacy matters 0621 update. https://pifonline.org.uk (4 January 2023, date last accessed).

[23] Berkman ND , Sheridan SL, Donahue KE, Halpern DJ, Crotty K. Low health literacy and health outcomes: an updated systematic review. Ann Intern Med 2011;155:97–107. 21768583 10.7326/0003-4819-155-2-201107190-00005

