# Supplementary material for: Digital exclusion as a potential cause of inequalities in access to care: a survey in people with inflammatory rheumatic diseases
Source: Rheumatol Adv Pract. 2023 Jan 6;7(1):rkac109. doi: 10.1093/rap/rkac109 (PMC9831060; doi:10.1093/rap/rkac109)
Supplement: rkac109_Supplementary_Data [file rkac109_supplementary_data.pdf]

Investigating the COVID Experience of People with Arthritis and their Care (ICEPAC)

INSTRUCTIONS FOR THIS QUESTIONNAIRE

- The aim of this questionnaire is to find out more about **you** and the impact of the COVID pandemic on your rheumatology condition.
- There are no right or wrong answers and no one will be able to identify you from your responses.
- Please answer all the questions (by putting a cross in the relevant box) unless the instructions ask you to do something else.
- If you are completing a paper questionnaire, please check that you have answered all of the questions and return it in the pre-paid envelope enclosed. **No stamp is needed**.
- If you have any questions, or need help completing this questionnaire, please telephone the ICEPAC research team on **07815 594071**

Thank you for your help with this study.

SECTION 1: Your inflammatory arthritis condition

1) Which of the following inflammatory conditions do you have?
(Please check all that apply)

Rheumatoid Arthritis (RA)	☐	Systemic Lupus Erythematosus (SLE or Lupus)	☐
Inflammation of the spine Ankylosing Spondylitis (AS) or axial spondylarthritis	☐	Other (please state below) ………...................	☐
Psoriatic Arthritis (PsA)	☐	None of the above	☐

2) Which of the following medicines do you take: (Please check only those that apply)

Hydroxychloroquine	☐	Anti-TNF treatment<br>e.g. Etanercept (Enbrel, Benepali), Infliximab, Adalimumab (Humira, Hyrimoz), Certolizimab (Cimzia)	☐
Methotrexate	☐	Other biologics (e.g. tocilizumab, rituximab, secukinumab, abatacept)	☐
Sulphasalazine	☐	Baricitinib, Tofacitinib, or Apremilast	☐
Leflunomide	☐	None of the above	☐
Prednisolone (steroid tablets)	☐		

3) a) If you answered yes to prednisolone do you take (1 tablet is normally 5mg):

5mg or less each day ☐	5 - 10 mg each day ☐	10 - 20mg each day ☐	Greater than 20mg each day ☐	N/A ☐

b) Have you been taking prednisolone for more than 4 weeks?

Yes ☐	No ☐	Don’t know/Unsure ☐

4) Have you ever been treated for any of the following medical conditions:
(Please check any that apply)

Diabetes	☐	Ischaemic Heart disease (such as angina/heart attack)	☐
Chronic Lung conditions (such as Asthma, Emphysema, Pulmonary Fibrosis)	☐	High Blood Pressure (Hypertension)	☐
Chronic kidney disease	☐	None of the above	☐
Cancer (solid tumours (e.g. lung or breast) or blood cancer like Leukaemia or Lymphoma)	☐		

SECTION 2: Shielding and You

1. Were you sent a letter from the government advising you to shield during the pandemic?

Yes ☐	No ☐	Don't know/Unsure ☐

2. Was another household member (e.g. partner/child) advised to shield?

Yes ☐	No ☐	Don't know/Unsure ☐

<u>If you answered no to question 1 and 2 please go directly to SECTION 3</u>

1) For those answering <u>yes</u> to either question, did you stick to this?

Mostly ☐	Sometimes ☐	Rarely ☐	Always ☐

2) Did you feel that shielding affected your health in any way?

Yes ☐	No ☐	Don't know/Unsure ☐

If yes, please give details:

3) Did you or a family member receive a letter in February 2021 advising you that you were in the clinically vulnerable category?

Yes ☐	No ☐	Don't know/Unsure ☐

SECTION 3: General impact of the pandemic

We are keen to understand how the pandemic has affected you and your family.

1) How has the COVID pandemic impacted <u>you</u> with regard to:

Your general physical health	Not at all ☐	Slightly ☐	Moderately ☐	Severely ☐	Not applicable ☐
Your mental health/mood	Not at all ☐	Slightly ☐	Moderately ☐	Severely ☐	Not applicable ☐
Your arthritis symptoms	Not at all ☐	Slightly ☐	Moderately ☐	Severely ☐	Not applicable ☐
Your work/employment	Not at all ☐	Slightly ☐	Moderately ☐	Severely ☐	Not applicable ☐
Your money/finances	Not at all ☐	Slightly ☐	Moderately ☐	Severely ☐	Not applicable ☐

2) How has the COVID pandemic impacted <u>your family/household</u> with regard to:

General physical health	Not at all ☐	Slightly ☐	Moderately ☐	Severely ☐	Not applicable ☐
Mental health/mood	Not at all ☐	Slightly ☐	Moderately ☐	Severely ☐	Not applicable ☐
Work/employment	Not at all ☐	Slightly ☐	Moderately ☐	Severely ☐	Not applicable ☐
Money/finances	Not at all ☐	Slightly ☐	Moderately ☐	Severely ☐	Not applicable ☐

SECTION 4: COVID impact on your arthritis

1. **For the following symptoms please select the statement which best describes your symptoms over the last two weeks and then if you think this is better or worse than before the pandemic.**

**1. Pain/stiffness during the day** How severe was your usual joint or muscle pain and/or stiffness overall during the **day** in the last 2 weeks?	Not at all ☐	Slightly ☐	Moderately ☐	Fairly Severe ☐	Very Severe ☐
Compared to before the pandemic is this…?	Much worse ☐	Slightly worse ☐	Unchanged ☐	Slightly better ☐	Much better ☐
**2. Pain/stiffness during the night** How severe was your usual joint or muscle pain and/or stiffness overall during the **night** in the last 2 weeks?	Not at all ☐	Slightly ☐	Moderately ☐	Fairly Severe ☐	Very Severe ☐
Compared to before the pandemic is this…?	Much worse ☐	Slightly worse ☐	Unchanged ☐	Slightly better ☐	Much better ☐
**3. Walking** How much have your symptoms interfered with your ability to walk in the last 2 weeks?	Not at all ☐	Slightly ☐	Moderately ☐	Fairly Severe ☐	Very Severe ☐
Compared to before the pandemic is this…?	Much worse ☐	Slightly worse ☐	Unchanged ☐	Slightly better ☐	Much better ☐
**4. Washing/Dressing** How much have your symptoms interfered with your ability to wash or dress yourself in the last 2 weeks?	Not at all ☐	Slightly ☐	Moderately ☐	Fairly Severe ☐	Very Severe ☐
Compared to before the pandemic is this…?	Much worse ☐	Slightly worse ☐	Unchanged ☐	Slightly better ☐	Much better ☐
**5. Physical activity levels** How much has it been a problem for you to do physical activities (e.g. going for a walk or jogging) to the level you want because of your joint or muscle symptoms in the last 2 weeks?	Not at all ☐	Slightly ☐	Moderately ☐	Fairly Severe ☐	Very Severe ☐
Compared to before the pandemic is this…?	Much worse ☐	Slightly worse ☐	Unchanged ☐	Slightly better ☐	Much better ☐

**6. Work/daily routine** How much have your joint or muscle symptoms interfered with your work or daily routine in the last 2 weeks (including work & jobs around the house)?	Not at all ☐	Slightly ☐	Moderately ☐	Fairly Severe ☐	Very Severe ☐
Compared to before the pandemic is this…?	Much worse ☐	Slightly worse ☐	Unchanged ☐	Slightly better ☐	Much better ☐
**7. Social activities and hobbies** How much have your joint or muscle symptoms interfered with your social activities and hobbies in the last 2 weeks?	Not at all ☐	Slightly ☐	Moderately ☐	Fairly Severe ☐	Very Severe ☐
Compared to before the pandemic is this…?	Much worse ☐	Slightly worse ☐	Unchanged ☐	Slightly better ☐	Much better ☐
**8. Needing help** How often have you needed help from others (including family, friends or carers) because of your joint or muscle symptoms in the last 2 weeks?	Not at all ☐	Slightly ☐	Moderately ☐	Fairly Severe ☐	Very Severe ☐
Compared to before the pandemic is this…?	Much worse ☐	Slightly worse ☐	Unchanged ☐	Slightly better ☐	Much better ☐
**9. Sleep** How often have you had trouble with either falling asleep or staying asleep because of your joint or muscle symptoms in the last 2 weeks?	Not at all ☐	Slightly ☐	Moderately ☐	Fairly Severe ☐	Very Severe ☐
Compared to before the pandemic is this…?	Much worse ☐	Slightly worse ☐	Unchanged ☐	Slightly better ☐	Much better ☐
**10. Fatigue or low energy** How much fatigue or low energy have you felt in the last 2 weeks?	Not at all ☐	Slightly ☐	Moderately ☐	Fairly Severe ☐	Very Severe ☐
Compared to before the pandemic is this…?	Much worse ☐	Slightly worse ☐	Unchanged ☐	Slightly better ☐	Much better ☐
**11. Emotional well-being** How much have you felt anxious or low in your mood because of your joint or muscle symptoms in the last 2 weeks?	Not at all ☐	Slightly ☐	Moderately ☐	Severely ☐	Extremely ☐

Compared to before the pandemic is this…?	Much worse ☐	Slightly worse ☐	Unchanged ☐	Slightly better ☐	Much better ☐
**12. Understanding of your condition and any current treatment** Thinking about your joint or muscle symptoms, how well do you feel you understand your condition and any current treatment (including your diagnosis and medication)?	Completely ☐	Very well ☐	Moderately ☐	Slightly ☐	Not at all ☐
Compared to before the pandemic is this…?	Much worse ☐	Slightly worse ☐	Unchanged ☐	Slightly better ☐	Much better ☐
**13. Confidence in being able to manage your symptoms** How confident have you felt in being able to manage your joint or muscle symptoms by yourself in the last 2 weeks (e.g. medication, changing lifestyle)?	Completely ☐	Very well ☐	Moderately ☐	Slightly ☐	Not at all ☐
Compared to before the pandemic is this…?	Much worse ☐	Slightly worse ☐	Unchanged ☐	Slightly better ☐	Much better ☐
**14. Overall impact** How much have your joint or muscle symptoms bothered you overall in the last 2 weeks?	Not at all ☐	Slightly ☐	Moderately ☐	Very much ☐	Extremely ☐
Compared to before the pandemic is this…?	Much worse ☐	Slightly worse ☐	Unchanged ☐	Slightly better ☐	Much better ☐

1) **How many flares of your arthritis have you experienced between January 2020 and today**

0	1	2	3+

2) How did you manage these flares during this period? (Select all that apply)

Waited for flare to improve without treatment	☐
Took extra medicines such as painkillers or anti-inflammatories	☐
Used non-drug treatments (e.g. heat, exercise, splints)	☐
Consulted GP	☐
Consulted the Rheumatology Team or Helpline	☐
Went to A&E	☐

4) Taking medications for arthritis:

In the last month, compared to before the pandemic…

a) Have you needed more "as required" ("rescue" / "prn") doses of painkillers?

Yes ☐	No ☐	Don't know ☐

b) Have you needed increased doses of regular painkillers?

Yes ☐	No ☐	Don't know ☐

c) Have you lowered the dose of your arthritis medications?

Yes ☐	No ☐	Don't know ☐

d) Have you stopped any of your arthritis medications?

Yes ☐	No ☐	Don't know ☐

5) Since the start of the pandemic have any of the following changed?

	Decreased a lot	Decreased a little	Stayed the same	Increased a little	Increased a lot	Not applicable
Amount you sleep	☐	☐	☐	☐	☐	☐
Amount you smoke/vape	☐	☐	☐	☐	☐	☐
Amount of alcohol you drink	☐	☐	☐	☐	☐	☐
Time spent doing hobbies/things you enjoy	☐	☐	☐	☐	☐	☐
Amount of general physical activity you do	☐	☐	☐	☐	☐	☐
Amount of exercise you do	☐	☐	☐	☐	☐	☐
Amount of painkillers you take for arthritis	☐	☐	☐	☐	☐	☐
Your weight	☐	☐	☐	☐	☐	☐

SECTION 5: COVID Impact on your mood

1. **Over the <u>last 2 weeks</u> how often have you been bothered by the following problems? (Please check <u>ONE</u> box on each line)**

		Not at all	Several days	Over half the days	Nearly every day
a	Feel nervous, anxious or on edge..	☐	☐	☐	☐
b	Not being able to stop or control worrying.......................	☐	☐	☐	☐
c	Worrying too much about different things.........................	☐	☐	☐	☐
d	Trouble relaxing.......................	☐	☐	☐	☐
e	Being so restless that it is hard to sit still.................................	☐	☐	☐	☐
f	Becoming easily annoyed or irritable....................................	☐	☐	☐	☐
g	Feeling afraid as if something awful might happen.................	☐	☐	☐	☐

2. **Still thinking about the <u>last 2 weeks</u>, how often have you been bothered by any of the following problems? (Please put a cross in <u>ONE</u> box on each line)**

		Not at all	Several days	More than half the days	Nearly every day
a.	Little interest or pleasure in doing things........	☐	☐	☐	☐
b.	Feeling down, depressed, or hopeless...........	☐	☐	☐	☐
c.	Trouble falling or staying asleep, or sleeping too much...........	☐	☐	☐	☐
d.	Feeling tired or having little energy.................	☐	☐	☐	☐
e.	Poor appetite or overeating............................	☐	☐	☐	☐
f.	Feeling bad about yourself or that you are a failure or have let yourself or your family down.............	☐	☐	☐	☐
g.	Trouble concentrating on things, such as reading the newspaper or watching television........	☐	☐	☐	☐
h.	Moving or speaking so slowly that other people could have noticed. Or the opposite – being so fidgety or restless that you have been moving around a lot more than usual...	☐	☐	☐	☐

3. **Compared to before the pandemic do you think your mood is:**

Better ☐	Worse ☐	No different ☐

4. **For the following statements choose <u>ONE</u> of the following options:**

		**Hardly ever**	**Some of the time**	**Often**
**a**	How often do you feel that you lack companionship?	☐	☐	☐
**b**	How often do you feel left out?	☐	☐	☐
**c**	How often do you feel isolated from others?	☐	☐	☐

SECTION 6: COVID impact on accessing healthcare

We would like to understand more about how you accessed healthcare and information about arthritis during the pandemic.

1) To which of the following do you have access? (Select all that apply)

Landline telephone	☐	Desktop or laptop computer with internet access	☐
Basic mobile phone (phone calls and texts only)	☐	iPad or other tablet	☐
Smartphone (can access the internet)	☐		

2) Generally, do you use the internet (Please select **ONE** response):

Never	Sporadically (less than 1 day/week)	Regularly (1–3 days/week)	Frequently (4–6 days/ week)	Daily
☐	☐	☐	☐	☐

3) How often do you need to have someone help you when you read instructions, pamphlets, or other written material from your doctor or pharmacy?

Never	Rarely	Sometimes	Often	Always
☐	☐	☐	☐	☐

<u>**IF YOU DO NOT USE THE INTERNET PLEASE SKIP TO QUESTION 5**</u>

4) For the following statements regarding the confidence you have using the internet to help with your health, please indicate how strongly you agree or disagree.

	Strongly Disagree	Disagree	Neither agree nor disagree	Agree	Strongly Agree
a) I know how to find helpful health resources on the Internet	☐	☐	☐	☐	☐
b) I know how to use the Internet to answer my health questions	☐	☐	☐	☐	☐

c) I know what health resources are available on the Internet	☐	☐	☐	☐	☐
d) I know where to find helpful health resources on the Internet	☐	☐	☐	☐	☐
e) I know how to use the health information I find on the Internet to help me	☐	☐	☐	☐	☐
f) I have the skills I need to evaluate the health resources I find on the Internet	☐	☐	☐	☐	☐
g) I can tell high quality from low quality health resources on the Internet	☐	☐	☐	☐	☐
h) I feel confident in using information from the Internet to make health decisions	☐	☐	☐	☐	☐

5) During the pandemic, did you use any of the following to manage your arthritis:

Websites for information about arthritis (e.g. Versus Arthritis, NRAS, NASS)	☐	Appointment with your GP	☐
Haywood Telephone advice line	☐	Rheumatology Appointment	☐
Haywood email advice service	☐		

6) Rheumatology appointments

a) If you had a Rheumatology appointment was this appointment:

Telephone	☐	Video	☐	Face to Face	☐

b) Thinking about your most recent consultation was this:

Telephone	☐	Video	☐	Face to Face	☐

c) Was this consultation:

Routine	☐	Arranged urgently due to worsening of your condition	☐	Face to Face	☐

d) If routine, was your arthritis well controlled?

Yes ☐	No ☐	Unsure ☐

e) Were you given a choice whether your appointment was face to face or using telephone/video?

Yes ☐	No ☐	Unsure ☐

If you did not have a telephone/video consultation on your latest appointment please skip to part g)

f) Was your telephone/video appointment with a clinician that you knew or had met before?

Yes ☐	No ☐	Unsure ☐

g) For the following statements, please choose one of the options:

	Very Confident	Confident	Neither	Unconfident	Very Unconfident
How **confident** do you feel about talking to your rheumatology clinician on the telephone?	☐	☐	☐	☐	☐
How **confident** would you feel talking to your rheumatology clinician on a video call?	☐	☐	☐	☐	☐

	Very Satisfied	Satisfied	Neither	Unsatisfied	Very Unsatisfied
How **satisfied** do you feel about talking to your rheumatology clinician on the telephone?	☐	☐	☐	☐	☐
How **satisfied** would you feel talking to your rheumatology clinician on a video call?	☐	☐	☐	☐	☐

h) In the future, thinking about the following situations, would you prefer your appointment to be:

	Telephone	Video	Face to Face	To be given a choice at the time.
For a new/first appointment about a problem	☐	☐	☐	☐
For an urgent problem (e.g. arthritis flare)	☐	☐	☐	☐
For a routine review appointment	☐	☐	☐	☐

i) Is there anything else that you would like to tell us about your experience or views of telephone or video consultations?

SECTION 7: COVID symptoms

1) Do you think you have had COVID?

Yes ☐	No ☐	Unsure ☐

<u>IF NO PLEASE SKIP TO SECTION 8</u>

2) a) Did you have a COVID test?

Yes ☐	No ☐	Unsure ☐

b) If yes, was the result:

Positive ☐	Negative ☐

c) If you did test positive please state date (MM/YY):

M		M	Y	Y

3) Were you admitted to hospital with COVID to stay overnight?

Yes ☐	No ☐

If yes- for how many nights were you in hospital? ……………..nights

4) For each symptom, if relevant, please check the box for how long it lasted:

		How long it lasted				
	Not applicable	1-3 days	4-7 days	1-2 weeks	2-8 weeks	8 weeks+
**Fever or chills/shivering**	☐	☐	☐	☐	☐	☐
**New or Persistent Cough**	☐	☐	☐	☐	☐	☐
**Shortness of Breath**	☐	☐	☐	☐	☐	☐
**Loss or Alteration of Taste/Smell**	☐	☐	☐	☐	☐	☐
**Nausea, Vomiting or Diarrhoea**	☐	☐	☐	☐	☐	☐
**Body aches or Pain**	☐	☐	☐	☐	☐	☐
**Increased fatigue/tiredness**	☐	☐	☐	☐	☐	☐
**Headache**	☐	☐	☐	☐	☐	☐
**Chest pain**	☐	☐	☐	☐	☐	☐

5) Are you fully recovered from your COVID illness now?

Yes ☐	No ☐	Unsure ☐

IF YES SKIP TO QUESTION [9]

6) What problems do you notice that still trouble you after your illness? (check all that apply)

More short of breath than before illness	☐	More tired than before illness	☐
Muscle weakness (more than before)	☐	Cough (more than before)	☐
Chest pain	☐	Difficulty walking distances as well as before illness	☐
Brain fog/confusion	☐	Other: ………………………………………..	☐

7) Have you been told by a clinician that you may have "long COVID"?

Yes	☐	No	☐	Unsure	☐

8) a) Do you feel like you may have the condition "long COVID"?

Yes	☐	No	☐	Unsure	☐

b) If yes, is it difficult to separate from your arthritis symptoms?

Yes	☐	No	☐	Unsure	☐

9) Have you received a COVID vaccine?

Yes, Pfizer/BioNtech ☐	No, I haven't been offered a vaccine yet ☐
Yes, Oxford/AstraZeneca ☐	No, I didn't want to have a vaccine ☐
Yes, Other Vaccine ☐ ………………………………..	

10) Finally, is there anything else about how the COVID pandemic has affected you or your arthritis (positive and/or negative) that you would like to tell us?

SECTION 8: About You

This section contains some questions about you.

1. What is your date of birth? D D ☐☐ M M ☐☐ Y Y Y Y ☐☐☐☐

For example, if you were born on 6th April 1970, this would be entered as 06/04/1970

a) Are you:

Male ☐ Female ☐

3. Which ethnic group do you consider yourself to belong to?
(Please check **ONE** box only)

White	☐	Black/African/Caribbean/ Black British	☐
Asian/Asian British	☐	Mixed/multiple ethnic groups	☐
Other	☐	Prefer not to say	☐

4. What is your current employment status?
(Please check **ONE** box only)

Employed	☐	Retired	☐
Unemployed/seeking work	☐	Housewife/husband	☐
Not working due to ill health	☐	Other	☐

5. **If you are working,** what is your job title, or **if you are not working, or are retired,** what was your last job title?

____

6. **If you are working,** are you currently....
(Please put a cross in **ONE** box only)

Doing your usual job ☐	Doing lighter duties ☐
Working fewer hours ☐	On paid sick leave ☐
On paid annual leave/holiday ☐	On unpaid leave ☐

7. What is your current smoking status? (Please check **ONE** box only)

Never smoked	Previously smoked	Current smoker
☐	☐	☐

8. On average, how often do you drink alcohol? (Please check **ONE** box)

Daily or almost daily	3 or 4 times a week	Once or twice a week	1 to 3 times a month	Special occasions only	Never
☐	☐	☐	☐	☐	☐

9. What is your weight?

☐☐ stones ☐☐ lbs **or** ☐☐☐ kgs

10. What is your height?

☐ feet ☐☐ inches **or** ☐☐☐ cms

Thank you for completing this questionnaire.
